# Rapid divergence in vegetative morphology of a wind‐pollinated plant between populations at contrasting densities

**DOI:** 10.1111/evo.14539

**Published:** 2022-07-13

**Authors:** Jeanne Tonnabel, Patrice David, John R. Pannell

**Affiliations:** ^1^ Department of Ecology and Evolution University of Lausanne Lausanne CH‐1015 Switzerland; ^2^ CEFE, Univ Montpellier, CNRS, EPHE, IRD, Montpellier, France CNRS Montpellier 34293 France

**Keywords:** Experimental evolution, male‐male competition, polygamy, resource allocation, sexual dimorphism, sexual selection

## Abstract

Plant sexual dimorphism is thought to evolve in response to sex‐specific selection associated with competition for access to mates or resources, both of which may be density dependent. In wind‐pollinated plants in particular, vegetative traits such as plant size and architecture may influence resource acquisition and both pollen dispersal and receipt, with potential conflict between these two components of fitness. We evaluated the role of plant density in shaping plant traits by measuring evolutionary responses in experimental populations of the sexually dimorphic wind‐pollinated plant *Mercurialis annua*. After three generations of evolution, we observed divergence between high‐ and low‐density populations in several vegetative traits, whereas there was no divergence for reproductive traits. A reversal in the direction of sexually dimorphic traits expressed in young plants evolved in both low‐ and high‐density populations compared to the original population (stored as seeds). Compared to the source population, males at high density evolved to be taller when young, whereas at low density young females tended to become smaller. These results demonstrate that a simple change in plant density can induce age‐dependent and sex‐specific evolution in the ontogeny of vegetative organs, and illustrates the power of experimental evolution for investigating plant trait evolution.

Males and females of dioecious plants often differ in their morphological, life‐history, and physiological traits (Geber et al. 1999). Although sexual dimorphism in plants is rarely as extreme as that displayed by many animals (Lloyd and Webb 1977), it has nevertheless evolved multiple times during angiosperm diversification. The South African genus *Leucadendron* provides a striking example of morphological divergence between sexes with the degree of sexual dimorphism having evolved several times independently among species (Tonnabel et al. 2014). In other species, sexual dimorphism may differ significantly among populations (e.g., *Silene latifolia*, Delph et al. 2002; *Rumex hastatulus*, Puixeu et al. 2019), suggesting evolutionary divergence in response to spatial and temporal variation in selection experienced by the sexes. Plant density is one key factor that may vary dramatically among populations, and from one generation to the next. Density is particularly interesting in the context of sexual dimorphism because it should modulate the strength of competition both for mates and for resources.

Although the role of sexual selection in shaping plant evolution has been hotly debated (Stanton 1994; Grant 1995), this role is no longer controversial (Moore and Pannell 2011; Lankinen and Karlsson Green 2015). It is also possible in principle that sexual selection in plants might include a form of choice by females of their mating partners, but this remains poorly evaluated (Tonnabel et al. 2021). In contrast, the role of intrasexual competition among males or hermaphrodites to access sexual partners and fertilize ovules is now well established (Moore and Pannell 2011; Lankinen and Karlsson Green 2015). Indeed, both theory (Arnold 1994; Stanton 1994; Tonnabel et al. 2019a), and empirical work (Bond and Maze 1999; Delph and Herlihy 2012; Schiestl and Johnson 2013; Cocucci et al. 2014; Dorken and Perry 2017; Lankinen et al. 2017) demonstrates the importance of male‐male competition for trait evolution in plants, and for trait divergence between male and female plants (Tonnabel et al. 2019a, 2019b). Much of this work is consistent with Bateman's third principle, which posits that male reproductive success is more limited by the number of mates they have access to, and therefore by intrasexual competition for mates, than female reproductive success (Bateman 1948; Arnold 1994; Stanton 1994). Such differences between males and females in competition for mates—a classical prediction from sexual selection theory—have been validated in an angiosperm species (Tonnabel et al. 2019a) and in a moss species (Johnson and Shaw 2016).

Vegetative traits can also have a direct impact on the outcome of male‐male competition, particularly in wind‐pollinated plants in which both height and above‐ground plant architecture can affect pollen dispersal and receipt (Klinkhamer et al. 1997; Eppley and Pannell 2007; Pickup and Barrett 2012; Harder and Prusinkiewick 2013; Tonnabel et al. 2019a, 2019b). For example, variation in plant height, branching patterns, branch length, and canopy diameter may affect the release and dispersal of pollen grains (Klinkhamer et al. 1997; Harder and Prusinkiewick 2013). In the wind‐pollinated herb *Mercurialis annua*, either elongated inflorescences or longer branches have been found to promote pollen dispersal over greater distances, increasing the number of a male's mates (Eppley and Pannell 2007; Tonnabel et al. 2019a, 2019b). In the dioecious genus *Leucadendron*, evolutionary transitions from insect to wind pollination are strongly associated with strong sexual dimorphism in vegetative traits (Tonnabel et al. 2014; Welsford et al. 2016), perhaps as a result of sexual selection acting on male plant architecture, and because male and female morphologies can diverge without any risk of pollination failure owing to pollinator visiting only one sex (Vamosi and Otto 2002). Sexual selection should apply more to plant populations in which many males compete with one another to pollinate a limited pool of females than in populations where competition takes place among fewer males. As such, we should expect plant density to modulate the extent to which plant traits affect patterns of mating and thus the intensity of sexual selection.

Vegetative traits should not only have direct effects on mating success by influencing pollen dispersal, but they are of course also of primary importance in the acquisition of resources. On the one hand, numerous studies have shown that the two sexes have different reaction norms to resource availability (e.g., water or nutrients) by allocating resources to their organs differently (reviewed in Tonnabel et al. 2017). This difference suggests that differential costs of reproduction in males and females may translate into sex‐specific selection for accessing different resource components (Antos and Allen 1990; McDowell et al. 2000; Harris and Pannell 2008; Van Drunen and Dorken 2012). Differences between the sexes in their resource needs are likely to be especially important in wind‐pollinated plants, in which males produce large quantities of nitrogen‐rich pollen (Harris and Pannell 2008; van Drunen and Dorken 2012; Wright and Dorken 2014), whereas the production by females of seeds and fruits typically draws heavily on photosynthates and water (Antos and Allen 1990; McDowell et al. 2000; van Drunen and Dorken 2012). On the other hand, we should expect vegetative divergence between the sexes to be limited by a common need to maintain access to light and avoid losing the competitive race with neighbors, which affects multiple plant functions (Labouche and Pannell 2016). This limitation should apply less at low density, but at high density both males and females should adopt a similar architectural strategy (Labouche and Pannell 2016; Tonnabel et al. 2017). Thus, although males and females might differ in important ways in their needs for different resources, the extent to which they can afford to diverge will depend on the intensity of competition for light with neighbors.

Here, we explored the sex‐specific evolutionary responses to either low or high density in the wind‐pollinated dioecious annual herb *Mercurialis annua* using experimental evolution in ten populations over the course of three generations; Figure 1 provides a graphical summary of our experimental design and hypotheses. We followed classical procedures of experimental evolution, whereby potentially divergent selection is allowed to act under contrasting environmental conditions on the standing genetic variation sampled from a common founding population (Kawecki et al. 2012). In our case, the source population corresponds to an artificial population produced by open mating between plants originating from different populations; it is thus characterized by high genetic variance for several plant traits of interest, and integrates the variability that evolved in natural populations with variable densities, on average intermediate between our density treatments. Our previous analyses of the experimental populations indicate that the manipulated change in plant densities did elicit both stronger competition among males for accessing ovules at the higher density (Tonnabel et al. 2019a), and stronger competition for accessing light at the higher density (Tonnabel et al. 2017). Given that both sexual selection and competition for light might have sex‐specific effects, we first expected an overall difference between sexes in vegetative trait evolution when comparing the source population and the low‐ and the high‐density evolved populations (Fig. 1). We expected that the high‐density treatment should foster evolution of male traits involved in mating success (e.g., plant height, branch length, peduncle length; Tonnabel et al. 2019b), with intermediate values for the source population (Fig. 1). We also expected the high‐density treatment to foster the evolution of female traits typically involved in competition for light (e.g., plant height, branch length; Tonnabel et al. 2017), with intermediate values for the source population (Fig. 1).

**Figure 1:**
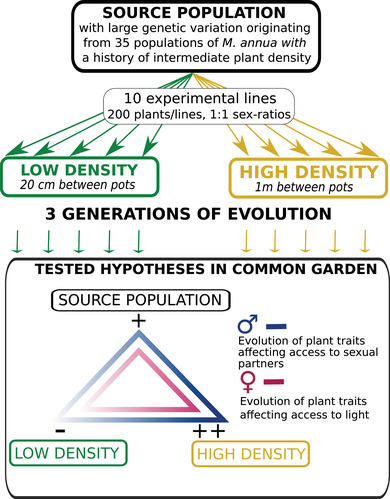
Summary of the experimental evolution protocol varying plant density in *Mercurialis annua* and of the tested hypotheses about the sex‐specific evolution of competitive traits in the common garden growing plants from the source population and high‐ and low‐density populations after three generation of evolution at the contrasted densities. Deeper colors indicates expectations of evolution of larger trait values.

## Materials and Methods

### STUDY SYSTEM AND SEED ORIGIN

*Mercurialis annua* is an annual wind‐pollinated herb distributed throughout southern and central Europe and around the Mediterranean Basin (Tutin et al. 1964). The species complex includes dioecious, androdioecious, and monoecious populations located in different parts of its range (Durand 1963; Pannell et al. 2004). For the current experiment, we focused on dioecious populations that naturally exhibit strong sexual dimorphism, with males being shorter than females and displaying stalk‐like (pedunculate) inflorescences that enhance pollen dispersal (Harris and Pannell 2008; Tonnabel et al. 2019b). Both sexes start producing flowers shortly after seed germination. Growth is indeterminate and reproduction continues until environmental conditions deteriorate, and plants die (Pannell 1997a).

We began our experiment by pooling seeds from 35 populations of *M. annua* sampled from northern Spain, with seed families sampled from approximately 30 females per population (see Tonnabel et al. 2017, 2019b and Fig. 1 for a summary of our experimental design). *Mercurialis annua* displays a metapopulation structure and dynamic characterized by frequent events of colonization and extinction (Obbard et al. 2006; Eppley and Pannell 2007); our experimental source population therefore represents the genetic and phenotypic variation present in wild populations at the metapopulation level. Before our experiment, we grew plants from the pooled source population in a common garden in Lausanne for three generations under uniform growing conditions (from 2012 to 2014) at an intermediate density compared to our later density treatments. This common garden aimed at reducing any maternal effects and/or genetic correlations caused by population subdivision across the metapopulation. We refer to seeds harvested after these three generations in the common garden as the source population. These seeds from the source population served two purposes in our experiment: (1) setting up our ten experimental populations with individuals drawn from a common pool and thus with a similar genetic composition, and (2) comparing the evolved traits in the low‐ and high‐density conditions to the source population under common environmental conditions in a common garden to test our evolutionary hypotheses (Fig. 1). three Preliminary results from another experiment with the same seed source found that seed storage did not affect seed germination rates in *Mercurialis annua* after a similar number of generations (N. Villamil‐Buenrostro and J. R. Pannell, unpubl. data), but we cannot rule out an effect on adult plant traits.

### EXPERIMENTAL EVOLUTION PROTOCOL

We established ten experimental populations of *M. annua*, divided into five populations that evolved independently from one another at low density and five others at a high density in seminatural conditions at the experimental field platform of the LabEx CeMEB in Montpellier, France (see Fig. 1 for a description of our experimental design). Each of the ten experimental populations were composed of 100 males and 100 females and were maintained at their assigned density for three generations (grown in spring of 2015, 2016, and 2017; see Tonnabel et al. 2017, 2019a,b for a description of the first generation), giving a total of 2000 plants grown each generation. Each garden consisted of a square array of 10 × 10 pots, each containing one male and one female growing together, and therefore competing for light (and other nutritive resources). Each of our experimental populations was established using seeds from the source population, following the classical approach adopted in studies of experimental evolution (Kawecki et al. 2012). We allowed plants to mate naturally in each of their populations (see below), then collected all seeds at the time of harvest, bulked the seeds of all females (within each population separately), and subsequently sowed seeds for the following generation by randomly sampling individuals from the bulked sample. This procedure ensured that each plant contributed, on average, to the following generation proportionally to the number of seeds it produced or sired.

At the beginning of each generation, seeds were individually germinated in greenhouses using pots filled with sterile compost. In the first generation, all seedlings came from our source population; in this first generation, seedlings were assigned to one of the ten experimental populations and these populations were kept separate from that point on. Prior to being transplanted into their populations in the field sites, seedlings were distributed randomly in space across the greenhouses and their positions were shuffled frequently. Seedlings were grown until plants could be sexed. At this point, after approximately 1.5 months of growth, pairs of males and females of the same experimental population were transplanted into 2 L pots of 20 cm of diameter containing sterile soil (1/3 of sieved clay and chalky soil, 1/3 of recycled compost, and 1/3 of compost). These male‐female pairs were moved outside into the garden, positioned in pots within their corresponding array. At this point, we continued growing all plants at a low density at a pot separation of 1.0 m. Approximately a month after transplanting the plants outside (with slight variation due to between‐year variation in weather conditions), we changed the position of all plants in the experimental populations. For the low‐density populations, we moved pots, but maintained their separation at 1.0 m. For the high‐density populations, pots were moved closer together, with an inter‐pot separation of 20 cm (between‐pot distances here and below designate distances between pot centers). Plants were allowed to continue to mate in their new positions for four weeks. After these four weeks, and for each population separately, we harvested all female plants and bulked them in drying bags. After drying all females separately for each population, we separated vegetative parts from seeds, which were kept to establish the next generation. As fruits disperse their seeds several days after fertilization, all seeds harvested after four weeks should have been fertilized during the phase of differential density application. Low adult mortality was observed in each generation.

Our experimental field consisted of two rows of five sites each. At each generation, we randomly assigned experimental populations among these ten sites by following two rules: we assigned (1) either two or three replicates of each treatment to each of the two rows and (2) each column contained one replicate of each treatment. This block design aimed at minimizing any differential effect of possible environmental gradients on populations belonging to the two different treatments. The 10 × 10 m sites were separated by 20 m to reduce gene flow between populations. Potential pollen flow was likely further reduced by the growth of dense vegetation in the meadow between sites (the growth of vegetation between pots within populations was prevented by a tarpaulin). Previous work on *M. annua* suggests that most mating occurs over short distances (Eppley and Pannell 2007; Hesse and Pannell 2011). We confirmed this small‐scale spatial pattern of mating on the basis of pollen dispersal kernels estimated for two of our experimental populations (Tonnabel et al. 2019b).

### ASSESSMENT OF EVOLUTIONARY RESPONSES TO SELECTION

After three generations of evolution, individuals from all experimental populations, plus those from the original source population, were established in a single common garden at the experimental field platform of the LabEx CeMEB in Montpellier, France. Seeds were initially germinated in greenhouses by adopting the growing procedures described above. When plants could be sexed, they were transplanted to the common garden in individual pots. The garden consisted of two blocks, one of 21 × 21 plants and the other of 20 × 22 plants, with an extra plant placed at one corner, giving a total of 441 plants per block. In both blocks, plants were grown in 2 L pots of 20 cm of diameter, placed with a between‐pot distance of 40 cm (a density that was intermediate between our two experimental densities). Some mortality occurred in the course of growth in the garden, which led to a dataset including 708 plants. Although this mortality prevented us from obtaining a fully balanced block design at the end of the experiment, all sexes and experimental populations were represented in each block, except for one population for which a manipulation error led to solely males being placed in both blocks (data available at https://doi.org/10.5061/dryad.sj3tx966n). We recorded the coordinates of each plant within the experimental blocks.

To assess male and female vegetative growth, we recorded plant height as the distance between the soil and the highest pair of leaves: (1) at the time of transplantation, (2) three weeks after transplantation, and (3) at the time of the final harvest (see below). In the following, we refer to our three repeated measures of plant height as “young,” “intermediate,” and “old.” For these plant height measurements, we excluded the length of exerted male peduncles to maintain comparability between the sexes. Three months after germination, all plants were harvested and measured. For all plants, we recorded its sex, the diameter of its canopy (as the longest horizontal length found between two leaves), the length of the first two branches (i.e., the lowest ramifications down the plant whose length we averaged and which value we refer to as branch length in the following), and its above‐ground vegetative dry biomass.

For males, we recorded the total number of pedunculate inflorescences, as well as their dry biomass, which we used as a proxy for pollen production (pollen accounts for 60% of male flower biomass; Pannell 1997b). On fresh male plants, we also measured the length of the five inflorescence‐bearing peduncles sampled on the fifth highest nodes of the primary axis. These five measures were later averaged, yielding our estimate of peduncle length. For females, we separated vegetative and reproductive tissues after drying the whole plant. We further weighed the seed mass and used an automatic seed counter (Elmor C3; Elmor Angewandte Elektronik, Schwyz, Switzerland) to assess seed number and size.

### STATISTICAL ANALYSIS

To test for sex‐specific evolution in each nonreproductive morphological trait, we fitted linear mixed models (LMMs), with sex, evolutionary treatment, and their interaction as fixed effects, and two random effects: (1) our two experimental blocks as implemented in the common garden, and (2) the experimental populations for which the random effect was treated as sex‐specific. Because reproductive traits are sex‐specific, the only fixed effect in the LMM was the evolutionary treatment (with blocks and population as random effects). Fixed effects in all these LMMs were tested using likelihood‐ratio tests (LRTs), a procedure that compares the fit of models with and without the effect of interest using a chi‐square statistic (Drton 2009). LRTs are particularly suited for comparing linear models with unbalanced designs, which is the case here, given that a single source population is compared with five independently evolved populations in each density treatment.

For nonreproductive traits, we first tested the sex × treatment interaction (representing differences in sexual dimorphism among treatments). When this interaction was significant, we divided the dataset into subsets to assess the treatment effect within each sex separately. When the interaction was not significant, we removed it from the model to test the treatment effect jointly in both sexes, and the sex effect jointly in all three treatments. These last tests inform on the presence of an evolutionary response aligned between sexes and on the presence of sexual dimorphism, respectively.

As the treatment × sex interaction and the treatment main effect compare three groups, we detailed the three possible pairwise contrasts in both cases (between source and low‐density, between source and high‐density, and between low‐ and high‐density)—to this end, we used LRTs on each contrast using datasets restricted to the two groups of interest. Both the main effect and the pairwise contrasts are referred to below as “treatment effects” to describe the statistical analyses performed. Among the many tests performed, some may be significant by chance, so we computed the expected number of false‐positive results at a 5% error rate.

We applied the same statistical procedure to the “reproductive effort,” computed as the biomass of the reproductive parts (i.e., seeds or male inflorescences) divided by the plant's vegetative biomass. Unless otherwise specified, we fitted all models using the R package “lme4” version 4_1.1‐21 (Bates et al. 2015) in R version 3.6.1 (R Core Team 2019). In addition, as spatial heterogeneity in conditions may have affected traits in our plots, we checked that the results were not changed when including a spatially autocorrelated error structure in the LMMs, as implemented in the spaMM package (Rousset and Ferdy 2014; see Method S1 for further details).

## Results

**Figure 2:**
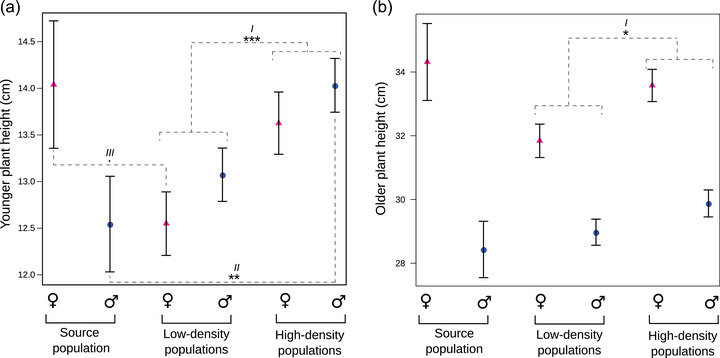
Predicted sex‐specific plant height in evolved and source populations of *Mercurialis annua* grown in a common garden after three generations of evolution. (a) Younger plant height and (b) older plant height were treated as response variables in our null models, which included both block and sex by population random effects. Females and males are represented by pink triangles and blue circles, respectively. The significance of differences between treatments (source, low‐density, and high‐density) in models combining both sexes and in sex‐specific models was evaluated using LRTs (˙*P* < 0.10, ^**^ *P* < 0.01, ^***^ *P* < 0.001). Separate models between sexes were performed only for the younger plant height (for which the sex by treatment interaction was significant). Horizontal bars indicate standard errors in model estimates. Roman numerals designate the different comparisons between treatments in each sex that are described in the *Results* section.

After three generations of evolution, plants that evolved under high density diverged from those evolved under low density for several vegetative traits that involved greater allocation to growth, as revealed by the pairwise contrasts (Table 1; e.g., comparison within Fig. 2b). These responses were common to both sexes (Table 1; e.g., comparison within Fig. 2b). In particular, both males and females that evolved at high density displayed greater plant height (at all ages) and greater canopy diameters than plants at low density (Tables 1 and 2; e.g., comparisons *I* in both Figs. 2a and 2b). In contrast, there was no evolutionary divergence among treatments for plant biomass or for reproductive effort (Table 1), nor were there any treatment differences for reproductive traits measured separately on males and females (i.e., peduncle length, number of peduncles, number of peduncles above the plant, peduncle mass, seed number, seed size, total seed mass; Table S1).

**Table 1:** Testing for (a) spatial structure in plant vegetative traits, (b) sex‐specific evolutionary response, and (c) non‐sex‐specific evolutionary response in these vegetative traits of *Mercurialis annua* plants that evolved at high versus low density and compared to our source population (SP) over the course of three generations, as assessed in a common garden. Both the main effect testing for an overall difference between treatment types (i.e., comparing the source vs. high‐ vs. low‐density populations) and the tests of pairwise contrasts are provided. Given the number of statistical tests reported for the vegetative traits dataset and an error rate of 5%, we expect that 3.3 tests on average should correspond to falsely significant results

Plant Trait	(a) Spatial Structure		(b) Sex × Treatment Effect								(c) Treatment Effect							
			Main Effect		Pairwise Contrasts						Main Effect		Pairwise Contrasts					
	(df = 3)		SP – High – Low (df = 2)		SP – High (df = 1)		SP – Low (df = 1)		Low – High (df = 1)		SP – High – Low (df = 2)		SP – High (df = 1)		SP – Low (df = 1)		Low – High (df = 1)	
	*χ* ^2^	*P*	*χ* ^2^	*P*	*χ* ^2^	*P*	*χ* ^2^	*P*	*χ* ^2^	*P*	*χ* ^2^	*P*	*χ* ^2^	*P*	*χ* ^2^	*P*	*χ* ^2^	*P*
Younger height ♂ ♀	6.07	0.11	**6.56**	**0.038**	**5.71**	**0.017**	**4.88**	**0.027**	0.118	0.73	**13.7** **8.00**	**0.0011** **0.018**	**7.46** 0.523	**0.0063** 0.47	1.35 3.45	0.24 0.063	**28.9**	**<0.0001**
Intermediate height	**20.4**	**0.00014**	3.34	0.19	1.33	0.25	2.67	0.10	3.34	0.19	**7.13**	**0.028**	0.114	0.74	0.661	0.42	**7.13**	**0.028**
Older height	**15.6**	**0.0013**	3.68	0.16	2.07	0.15	2.15	0.14	1.02	0.31	**6.83**	**0.033**	0.530	0.47	0.230	0.63	**6.10**	**0.014**
Canopy diameter	**8.27**	**0.041**	0.266	0.88	0.224	0.64	0.297	0.59	0.0665	0.80	**6.08**	**0.048**	1.47	0.23	7.00×10^‐4^	0.98	**5.49**	**0.019**
Branch length	**10.8**	**0.013**	1.12	0.57	0.873	0.35	1.15	0.28	0.0570	0.81	**7.52**	**0.023**	2.73	0.099	**5.05**	**0.025**	3.46	0.063
Biomass	**16.8**	**0.00079**	0.891	0.64	0.169	0.68	0.00390	0.95	0.859	0.35	3.71	0.16	2.98	0.084	3.80	0.051	0.0876	0.77
Reproductive effort	7.65	0.054	2.20	0.33	1.03	0.31	0.120	0.73	1.71	0.19	0.154	0.93	0.0957	0.757	0.0974	0.76	<0.0001	1.00

*Note*: The spatial structure for plant traits was evaluated by constructing models that explained them as a function of a spatial random effect modeled by a Matérn function, including three parameters. Models were fitted by maximum likelihood for performing LRTs between models differing in their fixed‐effects structure, and by restricted maximum likelihood for LRTs between models differing in their random‐effect structure. Significant *P*‐values are highlighted in bold and degrees of freedom (df) are provided for each type of LRT.

**Table 2:** Predicted sex‐specific vegetative traits (intermediate height, canopy diameter, biomass, and reproductive effort) in evolved and source populations (SP) of *Mercurialis annua* grown in a common garden after three generations of evolution

	Intermediate Height	Canopy Diameter	Biomass	Reproductive Effort
SP – Females	29.7 (±0.935)	11.5 (±0.681)	5.09 (±0.303)	0.0783 (±0.00969)
SP – Males	25.9 (±0.776)	9.71 (±0.541)	3.52 (±0.269)	0.170 (±0.00798)
Low – Females	27.6 (±0.439)	11.2 (±0.267)	4.80 (±0.207)	0.0831 (±0.00363)
Low – Males	26.0 (±0.405)	9.89 (±0.235)	3.20 (±0.202)	0.170 (±0.00315)
High – Females	29.1 (±0.428)	11.8 (±0.257)	4.74 (±0.206)	0.0884 (±0.00345)
High – Males	26.7 (±0.413)	10.4 (±0.244)	3.29 (±0.203)	0.166 (±0.00330)

*Note*: The null models predicted each response variable as a function of sex, treatment, and their interaction and included both block and sex by population random effects.

Plants from the low‐ or high‐density populations also diverged from the source population in two vegetative traits: young plant height and branch length (Table 1). The changes in young plant height differed between sexes to the extent of manifesting a reversal in the direction of dimorphism, as revealed by significant sex × treatment interactions (Tables 1 and 2; Fig. 2a). As a result, sexual dimorphism was not significantly different between low‐ and high‐density lines, but both differed from the source population (pairwise contrasts in the sex × treatment effect; Table 1). When young, females were taller than males in the source population, as is typical for *M. annua* (e.g., Harris and Pannell 2008), but the reverse pattern was found for the evolved populations of both the low‐ and high‐density treatments (Fig. 2a; sex effect: low‐density: *χ_i_* ^2^ = 7.62, df = 1, *P* = 0.006; high‐density: *χ_i_* ^2^ = 12.0, df = 1, *P* = 0.001). This reversal in the direction of sexual dimorphism can be attributed both to males from the high‐density populations evolving to be ∼1.5 cm taller than those from the source population (note the significant effect of the source vs. the high‐density origin in models considering only males; Table 1; comparison *II* in Fig. 2a) and to females from the low‐density treatment evolving to be ∼1.5 cm shorter than those from the source population (marginally significant effect of the source vs. low‐density treatments in models that included only females; Table 1; comparison *III* in Fig. 2a). Therefore, although both density treatments had similar sexual dimorphism, young plants of both sexes were taller in populations from the high density than in plants from the low‐density treatment (low‐high contrast in treatment effect; Table 1). Regardless of sex, plants that evolved at both densities also displayed shorter branches compared to the source population, but this difference was significant only for low‐density populations (see contrasts in branch length, treatment effect; Tables 1 and 2; Fig. S1).

## Discussion

Our experiment revealed rapid evolutionary responses to differences in plant density, with divergence in several vegetative traits between the two densities. After only three generations of divergent evolution, plants evolving at high‐density differed from those evolving at low‐‐density, being taller at all ages and displaying a wider canopy in both sexes. We also observed a reversal in the direction of sexual dimorphism in plant size in young plants in both evolved treatments compared to the source population. Our results show that sexual dimorphism in plant populations can evolve rapidly both in extent and direction, and illustrate the complex and unpredictable ways in which fitness through each of the two sexual functions maps to plant allocation and architectural phenotypes in the context of variation in density.

### INITIAL HYPOTHESES FOR THE SEX‐SPECIFIC EFFECTS OF DENSITY ON COMPETITIVE INTERACTIONS

We initially hypothesized that greater male‐male competition for accessing ovules in populations at high density would favor increased pollen production and male morphologies that promote effective pollen dispersal to mates. Such a prediction was motivated by our estimations of a 65% increase in the male opportunity for sexual selection (i.e., variance in the number of sexual partners; 0.43 vs. 0.26) in one high‐density population compared to another at low density, whereas this metric remained unaffected by the change in density for females (0.11 vs. 0.12; Tonnabel et al. 2019a). Of course, our hypothesis regarding male‐male competition rests on the assumption that mating patterns measured in the subsampled populations in the first generation (Tonnabel et al. 2019b) are representative of mating in the respective populations in generations two and three, too. Although we did not check this assumption throughout the experiment, the observed patterns of mating at high versus low density are consistent with expectations for leptokurtic pollen dispersal kernels that are commonly estimated for wind‐pollinated plants (Austerlitz et al. 2004; Goto et al. 2006; Gauzère et al. 2013; Geber et al. 2014), including *M. annua* (Tonnabel et al. 2019b).

Notwithstanding the effect of density on mating opportunities, previous results on the first generation (Tonnabel et al. 2017) also pointed to higher competition for light at high than low density. We therefore also hypothesized that greater competition for light in the higher density should particularly foster female morphologies that promote success in competition for light and thus for harvesting carbon, given that female reproduction relies more heavily on carbon than male function (Antos and Allen 1990; McDowell et al. 2000; Harris and Pannell 2008; van Drunen and Dorken 2012; Wright and Dorken 2014). Accordingly, in the first generation, the female opportunity for overall selection (i.e., variance in the number of offspring) was increased by 50% (0.48 vs. 0.32) in one high‐density population compared to another at low density, and this increment was not generated by competition for access to sexual partners nor by larger variance in access to light elicited by edge effects (Tonnabel et al. 2019a). Below, we discuss likely ways by which these two forms of competition (for mates acting mostly on males, and for resources acting predominantly on female plants) may have contributed to the evolutionary responses we observed, keeping in mind that our design ultimately does not allow us unambiguously to attribute each response to one or the other (as we manipulated only density).

### POSSIBLE RESPONSES TO SELECTION AT DIFFERENT DENSITIES VIA EFFECTS ON MATE VERSUS RESOURCE ACQUISITION

Some of our results are consistent with a response to selection on males for stronger access to mates. Plants mating at high density evolved greater height and canopy diameter than those mating at low density. Such responses are possibly the consequence of selection for mate acquisition by males, given that at least branch length has previously been shown to enhance pollen dispersal and mate acquisition in *M. annua* (Tonnabel et al. 2019b), and that plant height may affect pollen dispersal in general (Klinkhamer et al. 1997; Harder and Prusinkiewick 2013). The evolution of taller and larger plants at high density could alternatively be interpreted as a way to increase allocation to pollen production, as male flower number was strongly correlated with size traits (i.e., greater height, canopy diameter, and branch length) in our populations. However, this interpretation is not consistent with the lack of divergence in reproductive effort and reproductive traits between plants growing at high versus low density. Thus, differential selection on mate acquisition is a more plausible explanation than selection on pollen production to explain the differences among treatments, as was previously predicted in this system by estimates of both sexual and fecundity selection (Tonnabel et al. 2019b). Our results thus echo findings in animals that the intensity of sexual selection acting in each sex is affected by the density of individuals (Levitan 2004; Kokko and Rankin 2006).

Significantly, the evolution of larger canopy diameters and greater height for intermediate and older plants at high than at low density was not specific to males, that is, there was no indication of a divergence in sexual dimorphism between the two densities. Such a parallel response to differences in density by the two sexes would be consistent with expectations under strong genetic correlations for the relevant traits between the sexes, for example, with selection on males for more efficient pollen dispersal and a correlated response in females (a possible form of intralocus genetic conflict between the sexes; Hosken et al. 2019). Although sexual dimorphism at the juvenile stage did not diverge between high‐density and low‐density treatments in our experiment, it did change considerably compared to the source population (even to the extent of being reversed, albeit temporarily). This suggests that genetic correlations between the sexes are unlikely to have been much of a constraint on responses to selection in our experiment, at least at the juvenile stage, and they thus cannot easily explain the lack of difference in sexual dimorphism between the high‐ and low‐density populations at older ages. Assuming that intersexual genetic correlations did not pose a fundamental constraint on trait divergence between treatments in our experiment, it would seem that competition for light experienced by both sexes, or solely in females but combined with similar targets of selection than exerted by male‐male competition, may have contributed to the observed parallel evolution of larger vegetative traits at high density.

### EVOLUTION OF A REVERSAL IN SEXUAL SIZE DIMORPHISM BETWEEN THE TWO DENSITIES

Our experiment revealed a reversal in the direction of sexual dimorphism in plant size in young plants in both evolved treatments, compared to the source population. Given that our manipulations altered conditions experienced only later in life (recall that we imposed the density difference only after four weeks of growth), the expression of a response to selection by young plants may appear surprising. However, competitive ability late in life depends critically on resource allocation and physiological decisions taken much earlier, particularly because competition for light is strongly asymmetrical so that losing the competitive race early in life would have severe fitness implications later (Weiner 1990). Nevertheless, it remains difficult to find a single explanation for a reversal in the direction of sexual size dimorphism in young plants at both densities, given that we observed a decrease in young female plant height at low density versus an increase in young male plant height at high density. At low density, the evolution of shorter young females may reflect relaxed competition for light compared with the intermediate density experienced by the ancestral source population rather than differences in sexual selection, given that the number of sexual partners obtained by females was independent of density, at least in the first generation of the experiment (Tonnabel et al. 2019b). High density, by contrast, intensified (rather than relaxed) competition for mates specifically in males, to which populations may have responded by increasing growth in males (compared to the source population). Note that a male‐specific increase in allocation to growth is not likely to result from competition for light only, given reproduction in *M. annua* is thought to place heavier demands for carbon on females than males (Harris and Pannell 2008).

Our explanation resonates to some extent with observations made for the wind‐pollinated dioecious herb *Rumex hastatulus*. Pickup and Barrett (2012) and Puixeu et al. (2019) showed that males of *R. hastatulus* tend to be taller than females when pollen is dispersed, whereas females become the taller sex at the time of seed dispersal, consistent with a siring advantage of tall males. Similarly, in *M. annua*, the dispersal of pollen from inflorescences held above the plants or from longer branches has been shown to increase siring success (Eppley and Pannell 2007; Santos del Blanco et al. 2019; Tonnabel et al. 2019b). Although the timing of growth in height in *R. hastatulus* might seem to make more sense than our observations for *M. annua*, note that even a simple change in the competitive environment in *M. annua* could lead to age‐dependent changes in the direction of sexual dimorphism. To confirm the possibility that competition for mates late in a plant's life affects early resource allocation, future experiments should address the link between age‐dependent patterns of resource allocation and pollen dispersal (i.e., siring success).

### CORRESPONDENCE BETWEEN MEASURED SELECTION GRADIENTS AND RESPONSES TO SELECTION

It is interesting that the evolutionary responses observed after three generations of selection under contrasting density treatments did not in general align with the selection gradients that we had measured in the first generation, in which selection seemed to favor taller, broader, and heavier females at both densities (Tonnabel et al. 2019b). Although we found a difference in these female traits between the two density treatments, female size did not in fact increase compared to the ancestral population (rather, a decrease was observed in low‐density populations). Similarly, selection gradients in the first generation seemed to favor broader and longer branched males at high density and longer peduncles at low density, albeit weakly, and male height was not favored at either density (Tonnabel et al. 2019b). Yet our results indicate that male height did in fact evolve (especially at high density and at the juvenile stage). Similar inconsistencies have been found in other studies that compared selection gradients and the results of experimental evolution, and have been attributed to patterns of standing genetic variation, heritability, and pleiotropy (e.g., Gervasi and Schiestl 2017). Although strong heritabilities have typically been measured for plant height (e.g., Khan et al. 2018), artificial selection targeting this trait has also been found to drive changes in various other aspects of plant morphology, phenology, and physiology (Zu and Schiestl 2017). Unfortunately, we do not know the genetic variances and covariances for the traits we measured.

### CAVEATS

Some of our results may have been generated by the common changes in the conditions experienced by all populations relative to those experienced by the ancestors of our source population prior to the experiment's establishment. In particular, the fact that we harvested seeds at only one point in time and did not retain seeds for further generations that had been dispersed earlier likely gave rise to selection for late pollen and seed production, traits that may ultimately display genetic correlations with some of the traits we measured. Unmeasured maternal effects may also have impacted our results, not least because our evolved populations were cultivated for three generations in a different place and in different conditions than prevailed in the source population itself (although the latter had also grown in similar conditions for several generations). Nevertheless, it seems unlikely that these effects acted in a sex‐ or treatment‐specific manner. Finally, it is possible that differences in mating patterns between low‐ and high‐density conditions, as documented by Tonnabel et al. (2019b), may have led to differences in the importance of genetic drift between them. Although treatment‐specific genetic drift may ultimately compromise the interpretation of evolutionary responses (Kawecki et al. 2012), it seems unlikely to us that this effect would have strongly contributed to the differences observed here in only three generations, not least because the same census population sizes were maintained across time and populations, and because the differences in the mating patterns did not involve strong inbreeding, which would have had a greater impact on the effective size.

## Concluding Remarks

In summary, our experiment revealed rapid evolutionary changes in vegetative growth following evolution of a wind‐pollinated plant under contrasting densities, which occurred in only three generations. The results of our experiment join those of several others that demonstrate how responsive to selection experimental plant populations can be. These studies have focused on a number of traits related to plant reproduction, for example, floral scent production, the ability to self‐fertilize (Schiestl and Johnson 2013; Gervasi and Schiestl 2017; Ramos and Schiestl 2019), sex allocation (Dorken and Pannell 2009; Cossard et al. 2021), and pollen performance abilities (Lankinen et al. 2017). Our study now shows how a modification of growth conditions during the reproductive period may result in rapid changes to vegetative traits, too (and see Schiestl and Johnson 2013). Perhaps the most striking aspect of our findings is the demonstration of evolutionary responses in traits expressed early in a plant's life as a result of differences in reproductive success expressed at the time of reproduction, when seeds were sired by males and produced by females. As we have explained, it seems likely to us that responses by males and females were mediated by selection via competition for mates and for light, respectively. Finally, whether this interpretation is accurate or not, our results overall point to a complex interplay of different modes of selection that are evidently sensitive to a key demographic variable—plant density. Future studies might resolve some of this complexity through experiments that vary sex ratios or the number of pollen donors more directly that we have done here.

## AUTHOR CONTRIBUTIONS

JRP, PD, and JT designed the experiment. JT performed the experiment and statistical analyses. JT produced the first draft of the manuscript. All authors edited the manuscript.

## CONFLICT OF INTEREST

The authors declare no conflict of interest.

## DATA ARCHIVING

The dataset corresponding to trait evolution after three generations of growth at low versus high density is archived in Dryad Digital Repository (https://doi.org/10.5061/dryad.sj3tx966n).

Associate Editor: T. Giraud

Handling Editor: A. G. McAdam

## Supporting information


**Figure S1**. Predicted sex‐specific branch length in evolved and source populations of *Mercurialis annua* grown in a common garden after three generations of evolution. Branch length was treated as a response variable in our null models, which included both block and sex by population random effects. Females and males are represented by pink triangles and blue circles, respectively. The significance of differences between treatments (source, low‐density and high‐density) in models combining both sexes was evaluated using LRTs (*p < 0.05). Horizontal bars indicate standard errors in model estimates.

**Table S1**: Testing for (a) spatial structure in plant reproductive traits and (b) evolutionary response in these reproductive traits of *Mercurialis annua* plants that evolved at high‐ *versus* low‐density and compared to our source population (SP) over the course of three generations, as assessed in a common garden. Both the main effect testing for an overall difference between treatment types (i.e., comparing the source versus high‐ versus low‐density populations) and the effect of the pairwise contrasts are provided. Given the number of statistical tests reported for the vegetative traits dataset and an error rate of 5%, we expect that 1.1 tests on average should correspond to falsely significant results.
