# Supplementary material for: Rapid divergence in vegetative morphology of a wind‐pollinated plant between populations at contrasting densities
Source: Evolution. 2022 Jul 13;76(8):1737–48. doi: 10.1111/evo.14539 (PMC9544426; doi:10.1111/evo.14539)
Supplement: Supplementary file 1 — Figure S1. Predicted sex‐specific branch length in evolved and source populations of Mercurialis annua grown in a common garden after three generations of evolution. Branch length was treated as a response variable in our null models, which included both block and sex by population random effects. Females and males are represented by pink triangles and blue circles, respectively. The significance of differences between treatments (source, low‐density and high‐density) in models combining both sexes was evaluated using LRTs (*p < 0.05). Horizontal bars indicate standard errors in model estimates. [file EVO-76-1737-s001.docx]

**Supplementary Method S1**: accounting for spatial variation in the measured traits within the semi-natural common gardens

Because environmental variation occurring within our semi-natural common garden arrays could have elicited spatial variation in both vegetative and reproductive traits, we included in our LMMs an additional random effect describing the spatial distribution of the trait within the common garden array, implemented as a Matérn correlation function, which models autocorrelation as a function of distance between plants, fitted using the R package ‘spaMM’ version 2.6.39 (see Rousset & Ferdy, 2014 for further details). We then used LRTs to compare models with or without the spatial random effect; this analysis revealed that several traits were indeed subject to a spatial structure (Table 1). For these traits, we repeated the statistical procedure described in the main text, testing the effects of the treatment and treatment by sex interactions in models that included the effects of spatial structure. Because our results were robust to the inclusion of a spatial random factor explaining plant trait variation in LMMs for each of those variables that revealed significant spatial structure (see Table 1), we present only results of non-spatial LMMs as implemented in the R package ‘lme4’ (Bates *et al.*, 2015). We compared the statistical models either by maximum likelihood, or by restricted maximum likelihood when they differed in their fixed effects or in their random structure, respectively.

Rousset, F., and J-B. Ferdy. 2014. Testing environmental and genetic effects in the presence of spatial autocorrelation. Ecography 37:781-790.

Bates, D., M. Maechler, B. Bolker, S. Walker. 2015. Fitting linear mixed-effects models using lme4. Journal Statistical Software 67:1-48.
