# Supplementary material for: Rapid divergence in vegetative morphology of a wind‐pollinated plant between populations at contrasting densities
Source: Evolution. 2022 Jul 13;76(8):1737–48. doi: 10.1111/evo.14539 (PMC9544426; doi:10.1111/evo.14539)
Supplement: Supplementary file 2 — Table S1: Testing for (a) spatial structure in plant reproductive traits and (b) evolutionary response in these reproductive traits of Mercurialis annua plants that evolved at high‐ versus low‐density and compared to our source population (SP) over the course of three generations, as assessed in a common garden. Both the main effect testing for an overall difference between treatment types (i.e., comparing the source versus high‐ versus low‐density populations) and the effect of the pairwise contrasts are provided. Given the number of statistical tests reported for the vegetative traits dataset and an error rate of 5%, we expect that 1.1 tests on average should correspond to falsely significant results. [file EVO-76-1737-s002.docx]

**Table S1**: Testing for (a) spatial structure in plant reproductive traits and (b) evolutionary response in these reproductive traits of *Mercurialis annua* plants that evolved at high- *versus* low-density and compared to our source population (SP) over the course of three generations, as assessed in a common garden. Both the main effect testing for an overall difference between treatment types (i.e. comparing the source versus high- versus low-density populations) and the effect of the pairwise contrasts are provided. Given the number of statistical tests reported for the vegetative traits dataset and an error rate of 5%, we expect that 1.1 tests on average should correspond to falsely significant results.

|  | Plant trait | (a) Spatial structure | | (b) Treatment effect | | | | | | | | | | |
| --- | --- | --- | --- | --- | --- | --- | --- | --- | --- | --- | --- | --- | --- | --- |
|  |  |  | | Main effect | | | Pairwise contrasts | | | | | | | |
|  |  | (df=3) | | (1)  SP – High – Low  (df=2) | | | (2)  SP – High  (df=1) | | | (3)  SP – Low  (df=1) | | | (4)  Low – High  (df=1) | |
|  |  | χ^2^ | p | χ^2^ | p | χ^2^ | | p | χ^2^ | | p | χ^2^ | | p |
| Males | Peduncle length | 5.88 | 0.12 | 0.62 | 0.73 | 0.0218 | | 0.88 | 0.0718 | | 0.79 | 0.62 | | 0.43 |
|  | Number of peduncles | 3.41 | 0.33 | 0.496 | 0.78 | 0.330 | | 0.57 | 0.360 | | 0.55 | 0.0512 | | 0.82 |
|  | Number of peduncles on top | 5.85 | 0.12 | 3.20 | 0.20 | 3.38 | | 0.066 | 1.33 | | 0.25 | 0.603 | | 0.44 |
|  | Total peduncle mass | 7.77 | 0.051 | 1.64 | 0.44 | 1.34 | | 0.25 | 1.80 | | 0.18 | 0.0059 | | 0.94 |
| Females | Seed number | **11.6** | **0.00889** | 0.58 | 0.75 | 0.176 | | 0.68 | 0.542 | | 0.46 | 0.200 | | 0.65 |
|  | Seed size | 3.33x10^-9^ | 1.00 | 0.893 | 0.64 | 0.475 | | 0.49 | 0.934 | | 0.33 | 0.107 | | 0.74 |
|  | Total seed mass | **8.43** | **0.038** | 0.723 | 0.70 | 0.174 | | 0.68 | 9.00x10^-4^ | | 0.98 | 0.646 | | 0.42 |

**Notes:** The spatial structure for plant traits was evaluated by constructing models that explained them as a function of a spatial random effect modeled by a Matérn function, including three parameters. Models were fitted by maximum likelihood for performing LRTs between models differing in their fixed-effects structure, and by restricted maximum likelihood for LRTs between models differing in their random-effect structures. Significant p-values are highlighted in bold and degrees of freedom (df) are provided for each type of LRT.
